# Supplementary material for: Transient hysteresis and inherent stochasticity in gene regulatory networks
Source: Nat Commun. 2019 Oct 8;10:4581. doi: 10.1038/s41467-019-12344-w (PMC6783536; doi:10.1038/s41467-019-12344-w)
Supplement: Supplementary file 1 — Supplementary Information [file 41467_2019_12344_MOESM1_ESM.pdf]

Supplementary Information

Transient hysteresis and inherent stochasticity in gene regulatory networks

Pájaro, Otero-Muras et al.

Contents

1 Supplementary Notes 3

2 Supplementary Figures 5

1 Supplementary Notes

Supplementary Note 1

Correspondence between deterministic and stochastic counterparts. As it has been discussed in Pájaro *et al.*[1], the extreme states of a stationary bimodal distribution, namely those that include the highest and lowest probable states reached, satisfy:

$$-\rho(x) + \frac{-x}{ab(1-\varepsilon)} + \frac{a-1}{a(1-\varepsilon)} = 0, \tag{1.1}$$

where $\rho(x)$ is

$$\rho(x) = \frac{x^H}{x^H + K^H}. \tag{1.2}$$

Making zero the right hand side of equation

$$\frac{dx}{d\tau} = -x + abc(x), \tag{1.3}$$

and re-ordering terms, the set of all possible equilibria satisfies:

$$-\rho(x) + \frac{-x}{ab(1-\varepsilon)} + \frac{1}{(1-\varepsilon)} = 0. \tag{1.4}$$

Both expressions (1.1) and (1.4) are quite similar differing only in their respective last term of the left hand side, which become closer as $a \to \infty$, what implies large transcription rates as compared with protein degradation. This means that the most probable states of the microscopic system are near the stable equilibrium points described by the deterministic counterpart. Moreover, they become closer as the parameter a increases.

Supplementary Note 2

Mutual inhibitory gene regulatory motif in yeast[2, 3]. Let us define $\mathbf{x} = (x_1, x_2)$ with x_1 and x_2 being the amounts of Lacl and TetR respectively, and A be the amount of ATc. We use the following input functions to accommodate the network to the PIDE formulation [4]:

$$c_1(\mathbf{x}) = C_{rl} + \frac{k_t^{n_t}}{k_t^{n_t} + \left(x_2\left(1 + \frac{Ak_t}{k_{ATc}x_2}\right)^{-m}\right)^{n_t}}, \tag{1.5}$$

$$c_2(\mathbf{x}) = C_{rt} + r\frac{k_l^{n_l}}{k_l^{n_l} + x_1^{n_l}}, \tag{1.6}$$

where the parameters $n_t = 1.56$, $n_l = 3.35$, $k_t = 11$, $k_l = 264$, $k_{ATc} = 0.94$, and the degradation rate of the proteins $\gamma_x^i = 0.002\ \mathrm{min}^{-1}$ are taken from [3]. In [2] we find $C_{rl} = C_{rt} = 0.005$ and $n_t m \approx 11.5$, so we consider that $m \approx 7.37$. Finally we set $A = 4$ (because hysteresis was observed for a range of ATc between 0 and 250). We take γ_m^i, k_m^i and k_x^i such that $\dfrac{k_x^i k_m^i}{\gamma_m^i} = 1\ \mathrm{min}^{-1}$ for $i = 1, 2$. Finally, for slow dynamics (large burst frequency, i.e. high a values) we set $a_i = \frac{k_m^i}{\gamma_x^i} = 50$, obtaining $k_m^i = 0.1\ \mathrm{min}^{-1}$, $k_x^i = 0.4\ \mathrm{min}^{-1}$, $\gamma_m^i = 20\gamma_x^i = 0.04\ \mathrm{min}^{-1}$.

2 Supplementary Figures

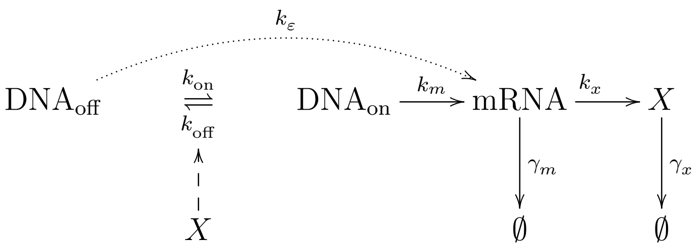

Supplementary Figure 1: Self-regulatory transcription-translation mechanism. The promoter is assumed to switch between active (DNA_{on}) and inactive (DNA_{off}) states, with rate constants k_{on} and k_{off} per unit time, respectively. The transition is assumed to be controlled by a feedback mechanism induced by the binding/unbinding of a given number of X-protein molecules. Transcription of messenger RNA (mRNA) from the active DNA form, and translation into protein X are assumed to occur at rates (per unit time) k_m and k_x, respectively. k_ε is the rate constant associated with transcriptional leakage. The mRNA and protein degradations are assumed to occur by first order processes with rate constants γ_m and γ_x, respectively.

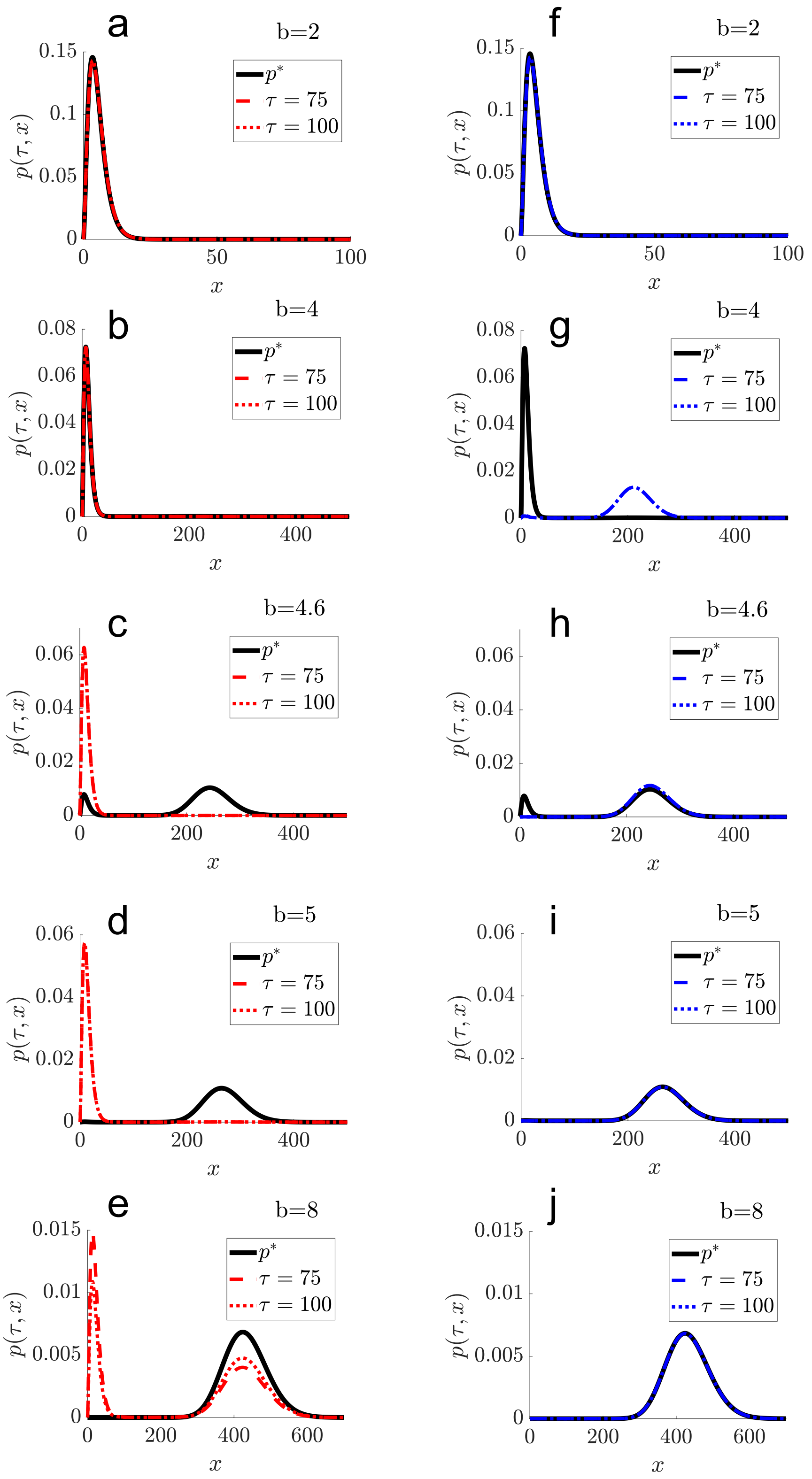

Supplementary Figure 2: Stationary and transient distributions obtained for different values of the b parameter ($a = 54$) for initial conditions $p(0, x) = \mathcal{N}(1, 0.1)$ (a,b,c,d,e) and $p(0, x) = \mathcal{N}(300, 1)$ (f,g,h,i,j). Transient distributions are represented by dashed ($\tau = 75$) and dotted ($\tau = 100$) lines. The black line is the stationary distribution.

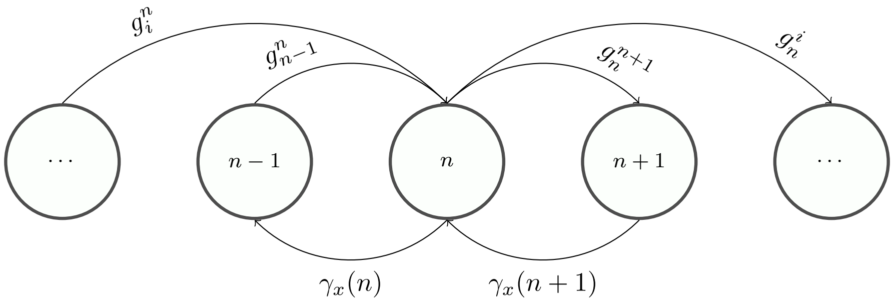

Supplementary Figure 3: Jump process representation of one protein produced in bursts, where one state n can be reached from lower states $0 \leq i < n$ with different transition probability functions g_i^n. Equivalently, from the state n the protein number can jump to higher states i with transition probability function g_n^i. The degradation follows a one step process (i. e. from state n to state $n-1$).

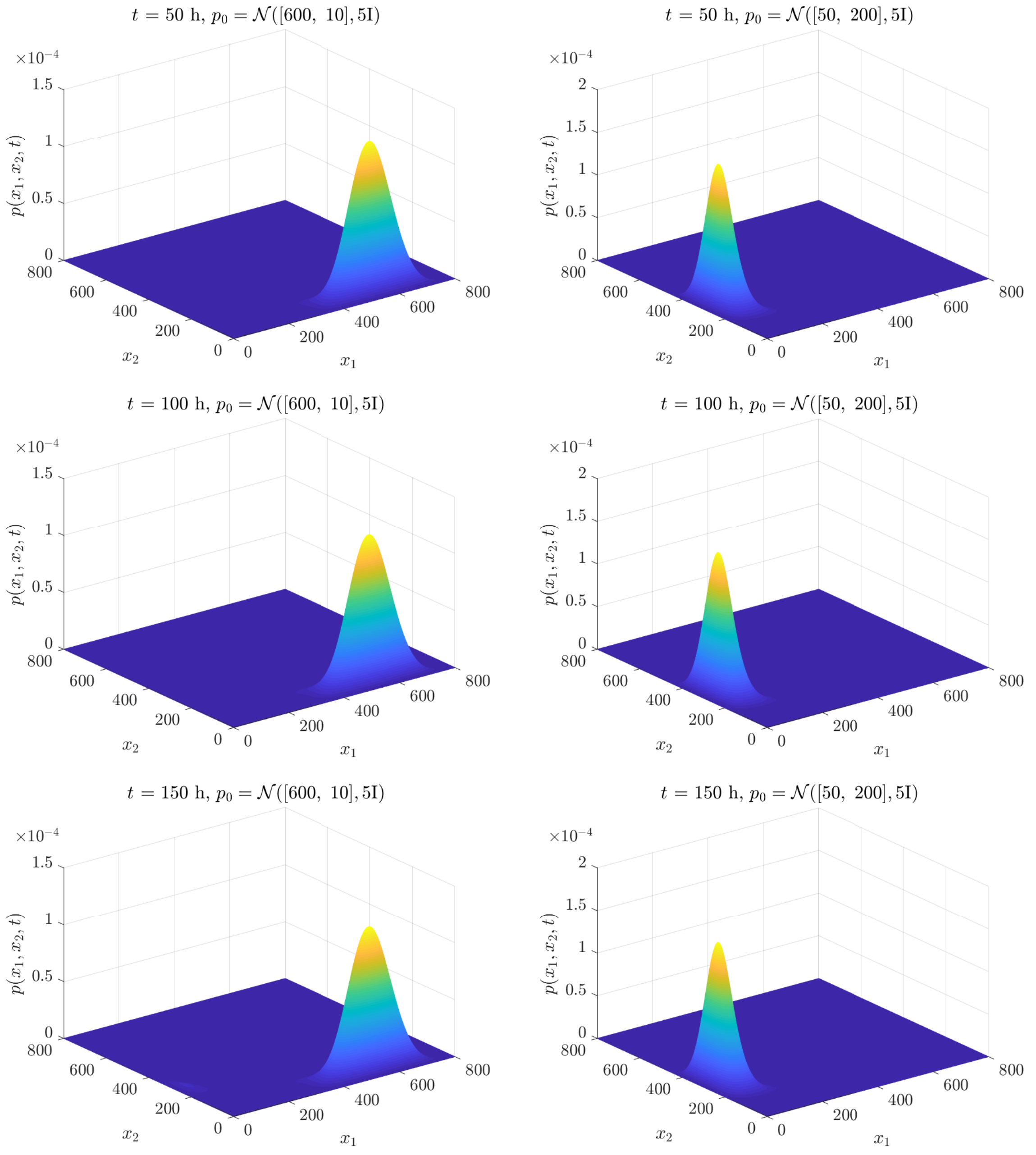

Supplementary Figure 4: Transient distributions of the LacI-TetR network with initial conditions $p_0 = \mathcal{N}([600,\ 10], 5\mathrm{I})$ (left column) and $p_0 = \mathcal{N}([50,\ 200], 5\mathrm{I})$ (right column). Initial conditions were chosen to be near the peaks of the stationary distribution.

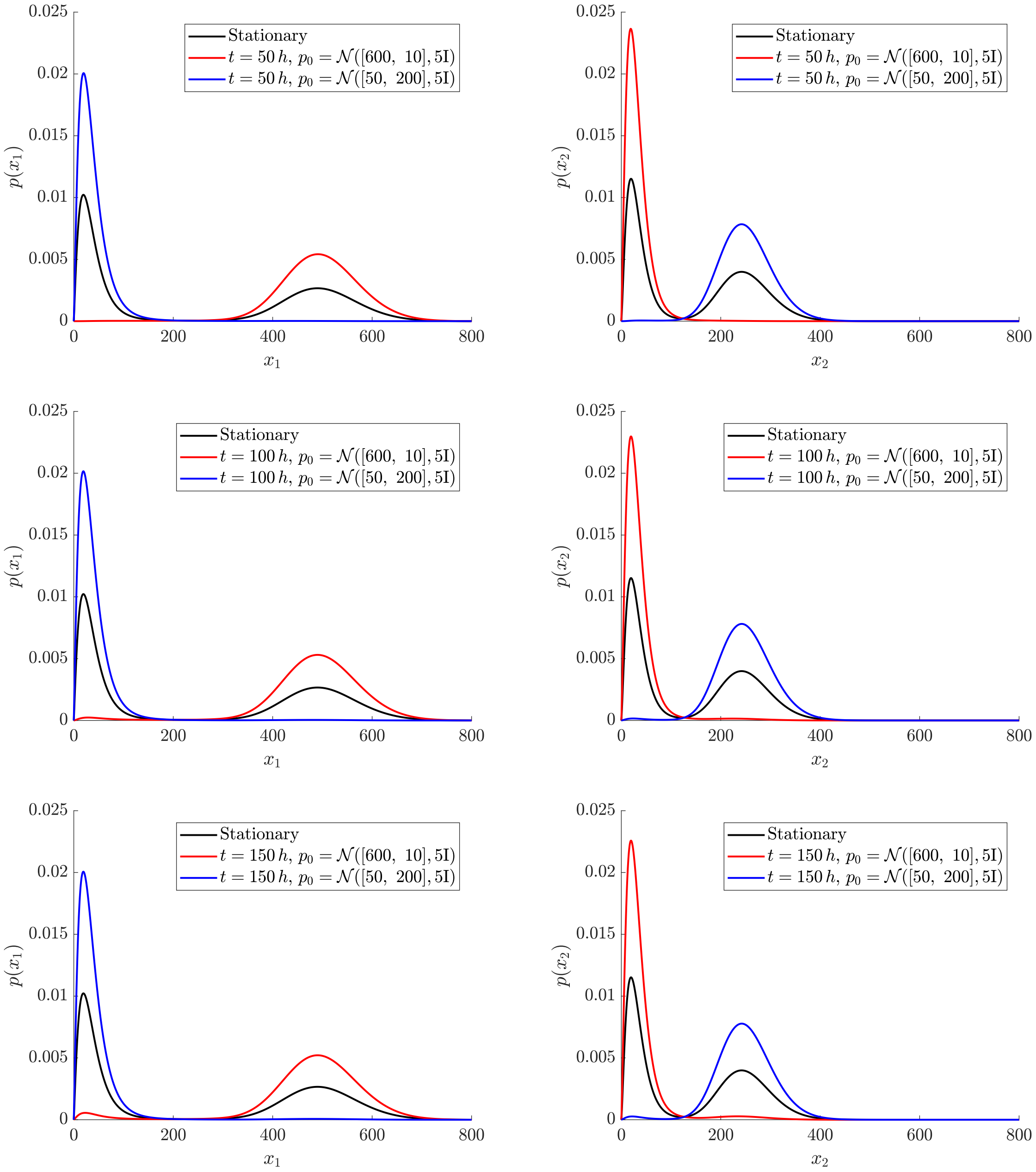

Supplementary Figure 5: Stationary (black lines) and transient marginal distributions of the LacI-TetR network with initial conditions $p_0 = \mathcal{N}([600,\ 10], 5\mathrm{I})$ (red lines) and $p_0 = \mathcal{N}([50,\ 200], 5\mathrm{I})$ (blue lines). Marginal distributions of LacI and TetR are depicted in the first and second columns, respectively.

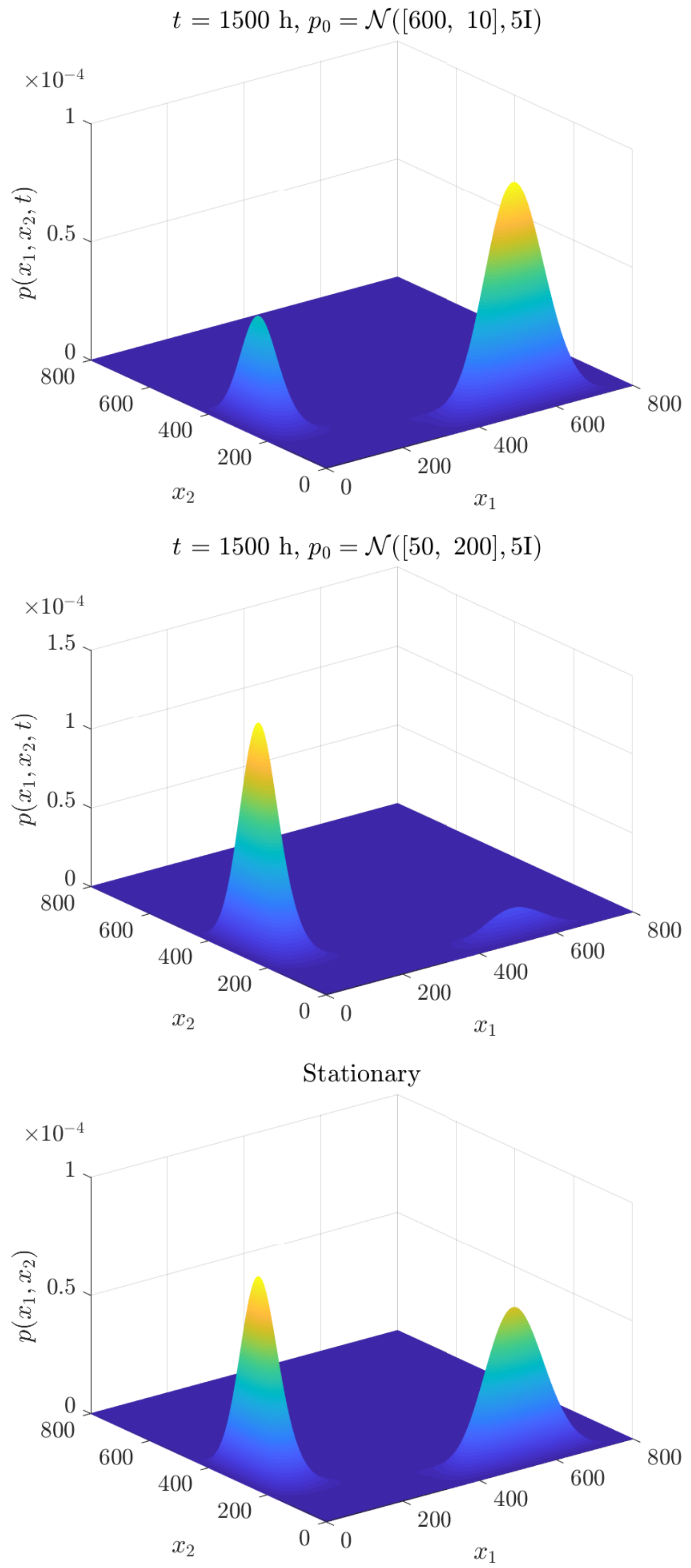

Supplementary Figure 6: Stationary (third row) and transient distributions (t = 1500 h) of the LacI-TetR network with initial conditions $p_0 = \mathcal{N}([600,\ 10], 5\mathrm{I})$ (first row) and $p_0 = \mathcal{N}([50,\ 200], 5\mathrm{I})$ (second row). Initial conditions were chosen to be near the peaks of the stationary distribution.

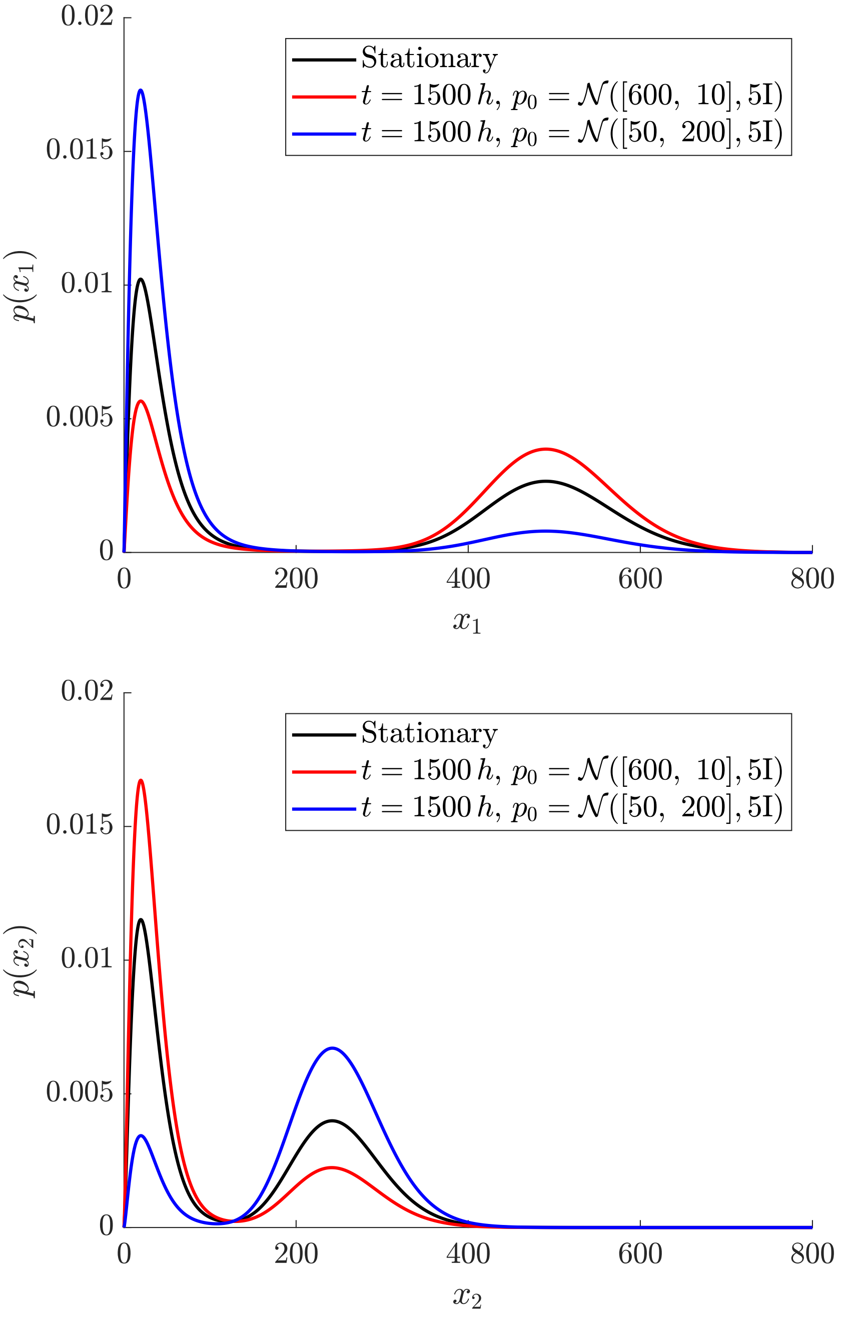

Supplementary Figure 7: Stationary (black lines) and transient marginal distributions ($t = 1500$ h) of the LacI-TetR network with initial conditions $p_0 = \mathcal{N}([600,\ 10], 5\mathrm{I})$ (red lines) and $p_0 = \mathcal{N}([50,\ 200], 5\mathrm{I})$ (blue lines). Marginal distributions of LacI and TetR are depicted in the first and second rows, respectively.

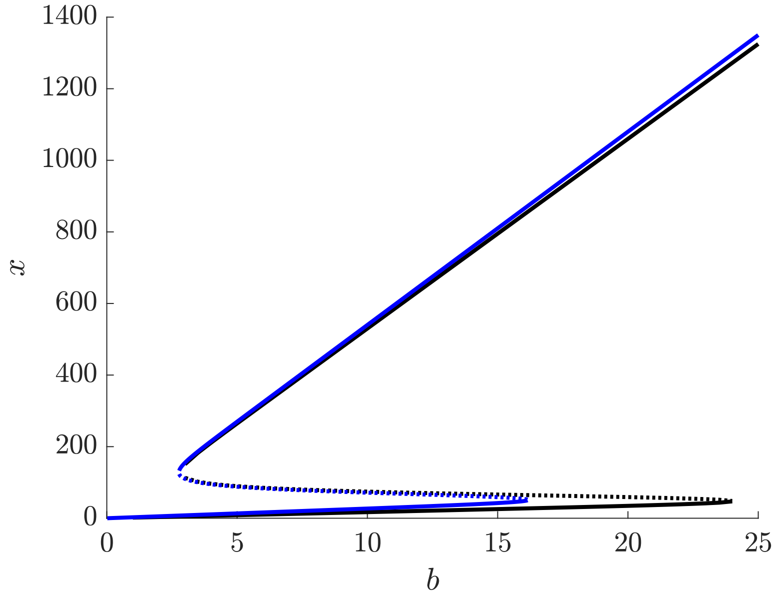

Supplementary Figure 8: Equilibrium states obtained from a deterministic representation (blue lines) as compared with the extremes (maxima and minimum) of the distributions that result from a stochastic description (black lines). Blue dotted lines correspond with unstable steady states whereas black dotted lines identify the minimum of the bimodal distribution.

Supplementary References

[1] M. Pájaro, A. A. Alonso, and C. Vázquez. Shaping protein distributions in stochastic self-regulated gene expression networks. *Phys. Rev. E*, 92(3):032712, 2015.

[2] T. Ellis, X. Wang, and J. J. Collins. Diversity-based, model-guided construction of synthetic gene networks with predicted functions. *Nat. Biotechnol.*, 27(5):465–471, 2009.

[3] M. Wu, R. Q. Su, X. Li, T. Ellis, Y. G. Lai, and X. Wang. Engineering of regulated stochastic cell fate determination. *Proc. Natl. Acad. Sci. U.S.A.*, 110(26):10610–10615, 2013.

[4] M. Pájaro, A. A. Alonso, I. Otero-Muras, and C. Vázquez. Stochastic modeling and numerical simulation of gene regulatory networks with protein bursting. *J. Theor. Biol.*, 421:51–70, 2017.
